# Establishment and Evaluation of a Rat Model of Sidestream Cigarette Smoke-Induced Chronic Obstructive Pulmonary Disease

**DOI:** 10.3389/fphys.2018.00058

**Published:** 2018-02-06

**Authors:** Genfa Wang, Nabijan Mohammadtursun, Jing Sun, Yubao Lv, Hualiang Jin, Jinpei Lin, Lingwen Kong, Zhengxiao Zhao, Hongying Zhang, Jingcheng Dong

**Affiliations:** ^1^Department of Integrative Medicine, Huashan Hospital, Fudan University, Shanghai, China; ^2^The Institutes of Integrative Medicine, Fudan University, Shanghai, China; ^3^Department of TCM, The Second Affiliated Hospital of Nanchang University, Nanchang, China; ^4^College of Xinjiang Uyghur Medicine, Hotan, China

**Keywords:** chronic obstructive pulmonary disease, passive smoking, cigarette smoke, rat model, airway remodeling

## Abstract

Chronic obstructive pulmonary disease (COPD) is a common cause of mortality worldwide. The current lack of an animal model that can be established within a certain time frame and imitate the unique features of the disease is a major limiting factor in its study. The present study established and evaluated an animal model of COPD that represents the early and advanced stage features using short-, middle-, and long-term sidestream cigarette smoke (CS) exposure. One hundred and nine Sprague–Dawley rats were randomly divided into 10 groups for different periods of sidestream CS exposure or no exposure (i.e., normal groups). The rats were exposed to CS from 3R4F cigarettes in an exposure chamber. Histological analysis was performed to determine pathological changes. We also conducted open-field tests, lung function evaluations, and cytokine analysis of the blood serum, bronchoalveolar lavage fluid, and lung tissue. The lung tissue protein levels, blood gases, and were also analyzed. As the CS exposure time increased, the indicators associated with oxidative stress, inflammatory responses, and airway remodeling were greater in the CS exposure groups than in the normal group. At 24 and 36 weeks, the COPD model rats displayed the middle- and advanced-stage features of COPD, respectively. In the 8-week CS exposure group, after the CS exposure was stopped for 4 weeks, inflammatory responses and oxidative responses were ameliorated and lung function exacerbation was reduced compared with the 12-week CS exposure group. Therefore, we established a more adequate rat model of sidestream CS induced COPD, which will have great significance for a better understanding of the pathogenesis of COPD and drug effectiveness evaluation.

## Introduction

Chronic obstructive pulmonary disease (COPD), a complex inflammatory disease, involves the destruction of the lung parenchyma and chronic inflammation of the peripheral airways, which leads to progressive airway narrowing and dyspnea (Lamprecht et al., 2011; Song et al., 2016). COPD has posed a substantial health care burden on many countries for centuries. It is the third leading cause of global death, based on the most recent figures from the Global Burden of Disease Study (Lozano et al., 2012; McLean et al., 2016). Its pathogenesis has not been fully elucidated, although an increasing number of patients have the disease. New therapeutic approaches have emerged but cannot fully counteract the progressive decline in lung function, which leads to disability and death, because the mechanisms underlying COPD are not well understood.

Animal models have been a key factor in forming objective views regarding the pathogenesis of COPD. However, mimicking the disease with its distinctive features within a certain time frame in that model is a major limiting factor for studying COPD (Beckett et al., 2013). Nevertheless, an animal model offers the best approach for studying all chronic diseases, such as COPD (Canning and Wright, 2008).

Cigarette smoking is the leading cause of most cases of COPD; approximately one in five chronic smokers develop COPD (Taylor, 2010). Cigarette smoking increases oxidative stress in the lungs and induces local inflammation, which affects the oxidant-antioxidant balance and causes airway remodeling and narrowing. Damage to the lung parenchyma leads to alveolar damage and emphysema, which may be major driving factors for the disease (Barnes, 2014; Wiegman et al., 2015).

Recent studies have reported that passive smoking is also harmful to the lungs, causing lung destruction through the introduction of toxic chemicals and oxidants to the lung, as well as inhibiting the repair pathways of the lungs (Birru and Di, 2012). It has been reported that 35% of female non-smokers, 33% of male non-smokers, and 40% of children worldwide are passive smokers (Oberg et al., 2011). Therefore, passive smokers are exposed to 50 times higher concentration of some chemicals than smokers themselves (Liu and Di, 2012). Moreover, most studies have focused on mainstream cigarette smoke (CS)-induced COPD animal models. However, an animal model that recapitulates the distinctive features of passive smoking is lacking.

To date, various methods of induction have been used to establish a COPD animal model for this purpose, but they are inadequate. In the present study, our aim was to establish a rat model of sidestream CS exposure-induced COPD using short-, moderate-, and long-term CS exposure. We designed six groups with different exposure times for this purpose and compared them with controls to evaluate the effects of short-, medium-, and long-term sidestream CS exposure on establishing early and advanced stage COPD rat models.

## Materials and methods

### Reagents

Kentucky 3R4F reference cigarettes were purchased from the University of Kentucky (Lexington, KY, USA), the enzyme-linked immunosorbent assay (ELISA) kits used in this study were purchased from R&D Systems (Minneapolis, MN, USA), and pentobarbital sodium was purchased from Merck (Darmstadt, Germany).

### Animals

Male Sprague-Dawley specific-pathogen-free rats (weight, 200 ± 20 g; age, 8 weeks) were purchased from Xipuer–Bikai Laboratory Animal Co., Ltd. (Shanghai, China; license number, SCXK [Hu]2008-0016), and 4–5 rats were housed per cage with *ad libitum* food and water. All rats were housed at a constant temperature (24°C) with a 12-h light/dark cycle. A total of 109 rats were randomly divided into 10 groups (8–12 rats/group). The normal group rats were exposed to normal air, while the model groups were exposed to CS. We tested six groups with 2-week (M1 group), 4-week (M2 group), 8-week (M3 group), 12-week (M4 group), 24-week (M6 group), or 36-week CS exposure (M7 group); one group with an 8-week CS exposure and a 4-week normal exposure (CS cessation; M5 group); and three normal groups of 12-week (N1 group), 24-week (N2 group), and 36-week (N3 group) no-smoke exposure. Animal group divisions are shown in Table 1.

**Table 1:** Animal group divisions.

**week**	**2 week**	**4 week**	**8 week**	**12 week**	**24 week**	**36 week**
**group**	**M1**	**M2**	**M3**	**N1,M4,M5**	**N2,M6**	**N3,M7**
	M1: 2-week cigarette smoke (CS) exposure group 1 (*n* = 12)					
	M2: 4-week CS exposure group 2 (*n* = 12)					
	M3: 8-week CS exposure group 3 (*n* = 12);					
	N1: 12-week normal group 1 (*n* = 8)					
	M4: 12-week CS exposure group 4 (*n* = 12)					
	M5: 8-week CS exposure + 4-week CS exposure cessation group (*n* = 12)					
	N2: 24-week normal group 2 (*n* = 8)					
	M6: 24-week CS exposure group 6 (*n* = 12)					
	N3: 36 -week normal group 3 (*n* = 9)					
	M7: 36-week CS exposure group 7 (*n* = 12)					

Procedures involving animals and their care were conducted in strict conformity with the national and local animal welfare bodies. All animal study protocols were approved by the Institutional Animal Care and Use Committee of Fudan University (Shanghai, China) and in conformity with the National Institutes of Health (China) guidelines.

### Animal model

The model was established according to references with minor modifications (Zheng et al., 2009). In brief, a group of rats were placed into a CS exposure chamber supplied with fresh air and sidestream smoke that was delivered to the chamber. For acclimation, model rats were exposed to a low dose of CS (2 cigarettes a day) in the first CS exposure week, and this was increased to 6 cigarettes a day at 15 min per cigarette with 5-min smoke free interval after each cigarette, with 3 CS exposures before noon and the other 3 after noon, 6 days per week. A standard smoke generator (Buxco, NC, USA) generated CS using 3R4F reference cigarettes. Early death was an exclusion criterion. Lung function tests were conducted before sacrifice. The 2-, 4-, 8-, 12-, 24-, and 36-week groups were sacrificed on days 15, 29, 57, 85, 169, and 253, respectively, for sample collection.

### Determination of lung function

All rats were anesthetized with an intraperitoneal injection of 2% pentobarbital (40 mg/kg), and tracheostomy was conducted, as previously described (Stevenson et al., 2005), with a standard catheter. All rats were tracheostomised and placed in a forced pulmonary maneuvre system (Buxco, NY, USA), which was connected to a computer. An average breathing frequency of 150 breaths/min was imposed on the anesthetized animals. Rats were forced to breathe against a closed valve at the mouth through which functional residual capacity (FRC) could be determined. During the quasi-static pressure-volume maneuvre, the lungs were inflated to a standard pressure of +30 cm H_2_O total lung capacity (TLC) and then slowly deflated until a pressure of −30 cm H_2_O was achieved. Different lung volumes, such as the forced expiratory volume in 1 s (FEV_1_), forced vital capacity (FVC), and maximal mid-expiratory flow(MMEF) were then determined.

### Lung histopathology

Lung tissue was dissected, fixed in 4% paraformaldehyde, embedded in paraffin, and stained with haematoxylin and eosin (H&E). The severity of inflammation was scored on a 0–5 scale, where 0 indicated normal; 1, mild inflammation with foci of inflammatory cells (minor); 2, a ring-shaped inflammatory cell infiltration with a one-cell-width layer (mild); 3, ring-shaped inflammatory cell infiltration with a two- to four-cell-width layer (moderate); 4, ring-shaped inflammatory cell infiltration with a four-cell-width layer (severe); and 5, severe inflammatory cell infiltration and no shape (very severe). Bronchus smooth muscle thickness, arteriolar smooth muscle thickness, and mean arteriolar wall area were also determined using a computer-aided medical image quantitative analysis system (Qiu Wei Biomedical Technology, Co., Shanghai, China). The destructive index (DI) and mean linear intercepts were measured according to a previous publication (Robbesom et al., 2003).

### Open-field test

Open-field tests were conducted 2 days before an animal was sacrificed. This test was used to evaluate locomotor activity and anxiety (Sudakov et al., 2013; Anchan et al., 2014). The experiments were conducted under dark and silent conditions. Rats were placed into an open-field box, and their behavioral patterns were measured. Their activity was analyzed, such as total distance traveled, rest time, center square entries, movement time, and residence time in the center and periphery.

### Blood gas analysis

To obtain valuable information on the extracellular acid–base status and gas exchange, the GEM Premier 3000 blood gas analyser (Instrumentation Laboratory, Lexington, MA, USA) was used to assay blood gas parameters. After the rats were anesthetized, blood samples were obtained and heparin sodium was inserted into the blood sample [25 IU/mL; Liu et al., 2014]. Ventilation patterns and pulmonary complications after upper abdominal surgery were determined by preoperative and postoperative computerized spirometry and blood gas analysis (Kum et al., 1996; Nie et al., 2012).

### Inflammatory mediators in bronchoalveolar lavage fluid and in the serum

Blood serum and bronchoalveolar lavage fluid samples were collected according to the method described in reference (Wei et al., 2016). In brief, blood samples preserved for 2 h at 4°C before centrifugation. Blood serum was collected by centrifuging the sample at 5,000 × g for 15 min. It was stored at −80°C before being analyzed. The left lung was subjected to lavage with 0.3 ml phosphate-buffered saline through the tracheal cannula to collect bronchoalveolar lavage fluid (BALF). The collected mixture was centrifuged at 1,000 × g for 10 min at 4°C, and the supernatant was preserved at −80°C. The pellet in the BALF was resuspended in phosphate-buffered saline (0.1 mL) for cell counting and analyzed using an automatic hematology analyser (BC-5300 Vet, Mindray Medical International, Ltd., Shenzhen, China). In the blood serum; BALF; lung tissue; cytokines such as interleukin (IL)-6, IL-8, IL-10, IL-1β, IL-17, tumor necrosis factor alpha (TNF-α), transforming growth factor beta-1 (TGF-β1), matrix metalloproteinase (MMP)-2, MMP-9, tissue inhibitor of metalloproteinase 1 (TIMP-1), malondialdehyde (MDA), superoxide dismutase (SOD), haeme oxygenase-1 (HO-1), and vascular endothelial growth factor(VEGF), were analyzed using an ELISA kit based on the manufacturer's instructions.

### Statistical analysis

Statistical analyses were performed using GraphPad Prism (Version 6.0; GraphPad Software, Inc., La Jolla, CA, USA) and SPSS 20.0 (IBM Corp., Armonk, NY, USA). All data are expressed herein as means ± standard error of the mean (SEM). A normal distribution test and test for equal variance was conducted for all data. The mean comparison between groups was conducted using analysis of variance (ANOVA), and a pairwise comparison was performed using the least significant difference test. The Kruskal–Wallis test was used to analyse data that were not normally distributed and did not have equal variance. The Mann–Whitney *U* test was used to assess significant differences in scalar or ordinal dependent variables. *p* < 0.05 was considered statistically significant.

## Results

### General condition and body weight changes

The control group rats were healthy. They excreted pale yellow urine and had glossy, smooth fur, bright eyes, stable breathing, full muscles, and quick responses. In the CS exposure groups, sneezing and coughing began after 4 weeks of CS exposure. As the exposure time increased, the rats had an accelerated respiratory rate, dyspnea, and their fur became dry and yellow. Breathing was slow and deep in some rats and fast and shallow in others. Physical activity declined, responses slowed, food and water intake decreased, and their urine became deep yellow.

**Figure 1:**
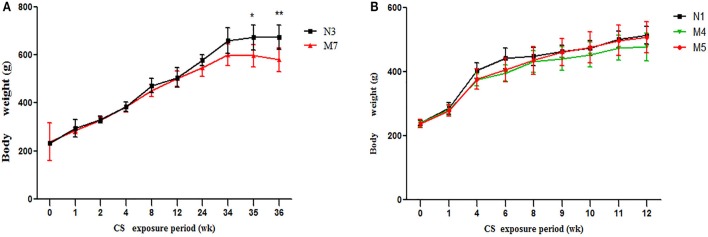
Body weight changes in the cigarette smoke (CS) exposure groups and normal groups. **(A)** The 36-week cigarette smoke exposure group (M7) and normal group (N3). **(B)** The 12-week cigarette smoke exposure group (M4), the 8-week CS exposure + 4-week recovery group (M5), and the 12-week normal group (N1). The values are presented as the means ± the standard error of the mean (SEM). ^*^p < 0.05 and ^**^p < 0.01 indicate a statistically significant difference compared to the control group.

Initial body weight was similar among all groups (*p* > 0.05). There was an upward trend in body weight gain in all groups. No significant differences occurred until 24 weeks of CS exposure (data not shown, *p* > 0.05). After 24 weeks of CS exposure, the body weight of the CS group rats displayed a declining trend, and this decline was significant in the 36-week model group (*p* > 0.01, Figure 1A), whereas the normal group showed a persistent body weight increase. This finding indicates that long-term passive smoking has negative effects on the body mass index of SD rats.

There were no significant changes in body weight gain between the 12-week (M4) CS group and 8-week CS exposure + 4-week CS cessation (M5) groups (*p* > 0.05). However, during the 4-week cessation period, the average body weight gain trend was higher in the M5 group than in the M4 group (Figure 1B).

### Pulmonary function changes

**Figure 2:**
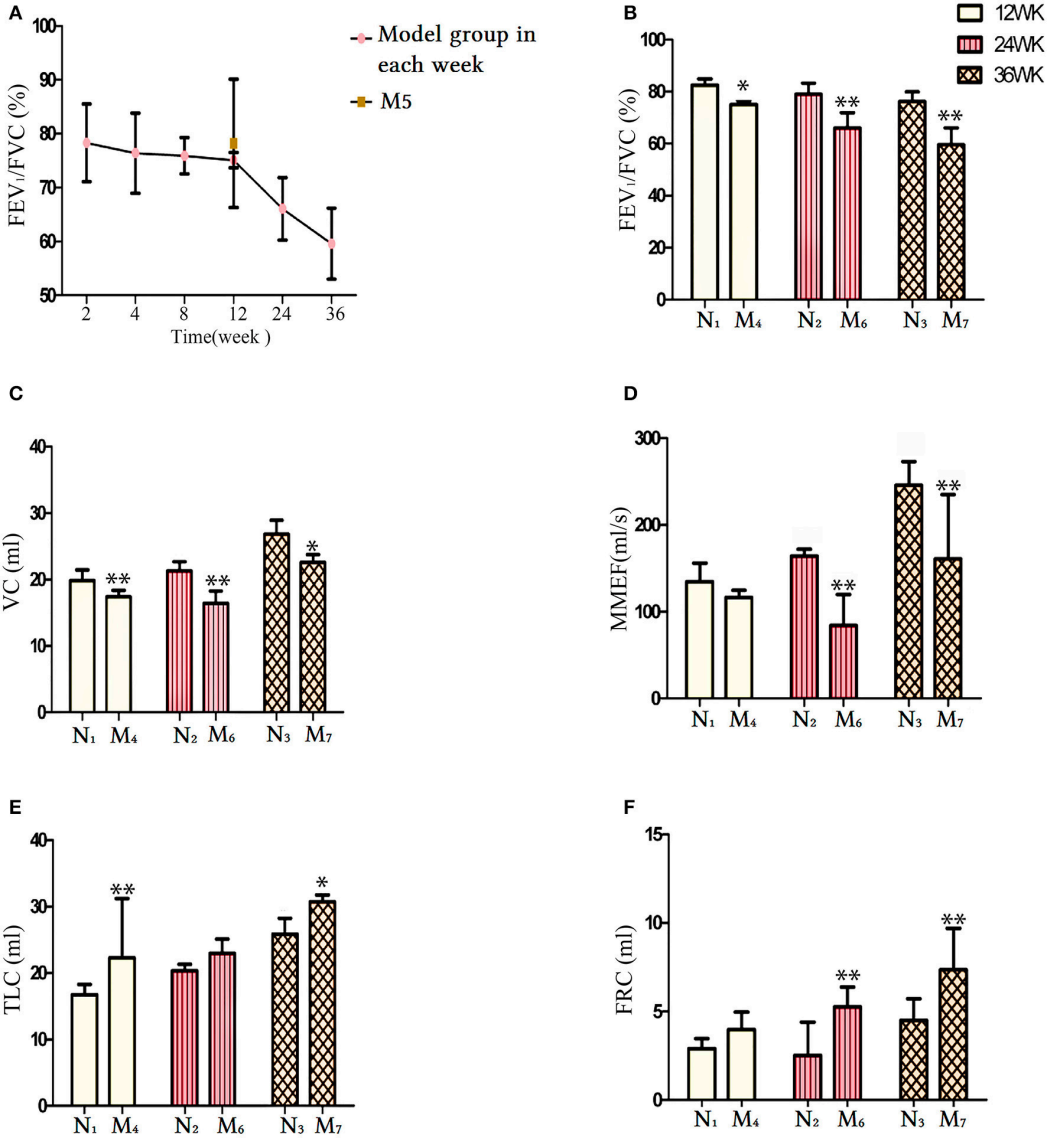
Comparison of lung function indicators between the normal groups (N1, N2, and N3) and their respective cigarette smoke (CS) exposure group. **(A)** The FEV1/FVC ratio in each CS exposure group. **(B)** The FEV1/FVC ratio at weeks 12, 24, and 36 in the CS exposure group. **(C)** The vital capacity (VC) level at 12, 24, and 36 weeks in the CS exposure group. **(D)** The MMEF level at 12, 24, and 36 weeks in the CS exposure group. **(E)** The TLC level at 12, 24, and 36 weeks in the CS exposure group. **(F)** The FRC level at 12, 24, and 36 weeks in the CS exposure group. The data are shown as the mean ± the standard error of the mean (SEM). ^*^p < 0.05 and ^**^p < 0.01 indicate a statistically significant difference compared to the control group. FEV1, forced expiratory volume in 1 s; FRC, functional residual capacity; FVC, forced vital capacity; MMEF, maximum mild expiratory flow; TLC, total lung capacity.

Lung function characteristics of the animals are presented in Figure 2. The FEV_1_/FVC ratio displayed a downward trend in all CS exposure groups (Figure 2A). Following 12-, 24-, and 36-week CS exposure, the FEV_1_/FVC ratio (Figure 2B) and VC (Figure 2C) significantly decreased compared with the same time periods in the control groups (N1, N2, and N3, respectively; *p* < 0.05 and *p* < 0.01, respectively). No significant differences were found in the maximal mild expiratory flow (MMEF) of the 12-week CS exposure group compared with the normal groups (*p* > 0.05). In contrast, significant decreases occurred in the 24- and 36-week CS exposure groups compared with the N2 and N3 groups, respectively (*p* < 0.01; Figure 2D). Indicators associated with emphysema were also investigated. TLC was increased in the model group after 12 and 36 weeks of CS exposure, compared with the N1 and N3 groups, respectively (*p* < 0.05 and *p* < 0.01, respectively), whereas no significant differences were seen at 24 weeks of CS exposure (*p* > 0.05; Figure 2E). FRC increased in the 24- and 36-week CS exposure groups (M6 and M7 groups, respectively) compared with the normal groups N2 and N3, respectively (*p* < 0.01; Figure 2F). This finding suggests the onset of pulmonary emphysema. There was little change in lung function indicators in the CS groups at 2, 4, and 8 weeks CS exposure compared with the control groups (*p* > 0.05, data not shown).

### Effects of CS exposure cessation on pulmonary function

**Figure 3:**
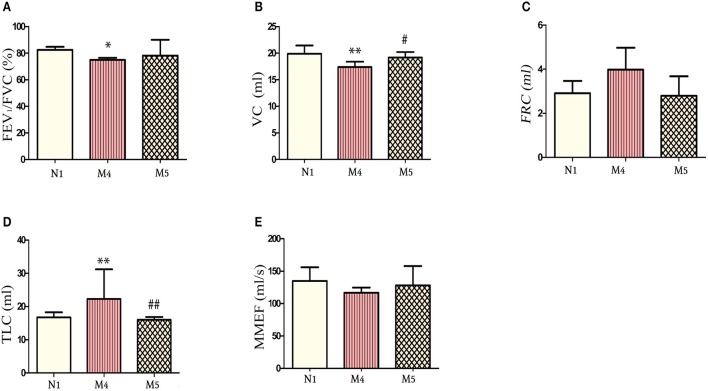
Comparison of lung function indicators between the normal group and the 12-week cigarette smoke (CS) exposure group and the 8-week CS exposure + 4-week CS exposure cessation group. **(A)** The FEV1/FVC ratio at 12 weeks in the normal group, M4 group, and M5 group. **(B)** The vital capacity (VC) at 12 weeks in the normal group, M4 group, and M5 group. **(C)** The FRC at 12 weeks in the normal group, M4 group, and M5 group. **(D)** The TLC at 12 weeks in the normal group, M4 group, and M5 group. **(E)** The MMEF at 12 weeks in the normal group, M4 group, and M5 group. The data are presented as the mean ± the standard error of the mean (SEM).*p < 0.05 and ^**^p < 0.01 indicate a statistically significant difference compared to the control group N1.^#^*p* < 0.05 and ^##^*p* < 0.01 indicates a statistically significant difference compared to the M4 group. FEV1, forced expiratory volume in 1 second; FRC, functional residual capacity; FVC, forced vital capacity; MMEF, maximal mild expiratory flow; TLC, total lung capacity.

As shown in Figure 3, the lung function of the CS cessation group was recorded. Comparisons of CS cessation group with the 12-week CS exposure group and the normal group (N1) revealed that the FEV_1_/FVC ratio was not changed (*p* > 0.05; Figures 3A) and VC were significantly increased compared to M4 group (*p* < 0.05; Figure 3B), whereas the TLC was decreased (*p* < 0.01, for both; Figure 3D). The FRC and MMEF were not significantly different between the normal groups and the M4 or M5 groups (*p* > 0.05; Figures 2C,E, respectively). These results indicate an improvement in pulmonary function in the M5 group and demonstrate that smoking cessation is one way to prevent exacerbation of COPD or to delay decreases in lung function.

### Pathological changes in rats

**Figure 4:**
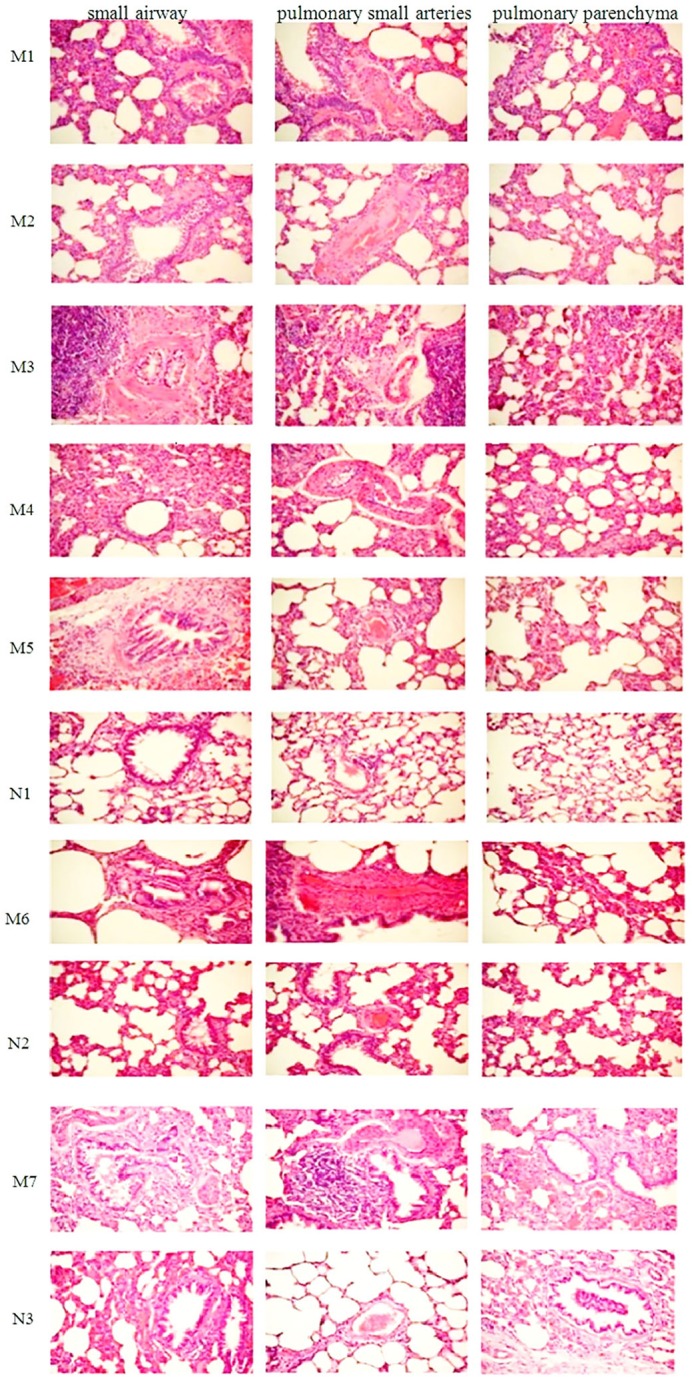
Photograph of HE-stained lung tissue under an optical microscope (×200). M1, 2-week cigarette smoke (CS) exposure group; M2, 4-week CS exposure group; M3, 8-week CS exposure group; M4, 12-week CS exposure group; M5, 8-week CS exposure + 4-week CS cessation group; M6, 24-week CS exposure group; M7, 36-week CS exposure group; N1, 12-week normal group; N2, 24-week normal group; N2, 36-week normal group. H&E, haematoxylin–eosin.

In the control group, few histological changes were observed in the lung tissue (Figure 4). In contrast, CS exposure in all groups (2–36 weeks) resulted in pathological changes to different extents. As shown in Figure 4, airway epithelial cell injury and mucus secretion from the bronchi, goblet epithelium cell metaplasia, inflammatory cell infiltration, and cell blocking in the bronchial lumen are evident. Emphysema, congestion, degeneration, and necrosis of epithelial cells increased with increasing CS exposure time (Figure 4; M1-M7). Epithelial and pulmonary architecture damage in the lungs, thickening of the basement membrane in airways, smooth airway muscle hypertrophy, enlargement of peripheral air space, and cell infiltration were also observed. Inflammatory cells were increased in the thickened alveolar interstitium. As the CS exposure time increased, the pathological condition became more aggravated, which peaked at 24 weeks (i.e., M6 group; Figure 4). At 36 weeks of exposure (i.e., M7 group), the lung tissue showed typical COPD pathological features (Figure 4). H&E staining indicated that a COPD model was successfully established.

### Quantitative analysis of lung tissue pathology

**Figure 5:**
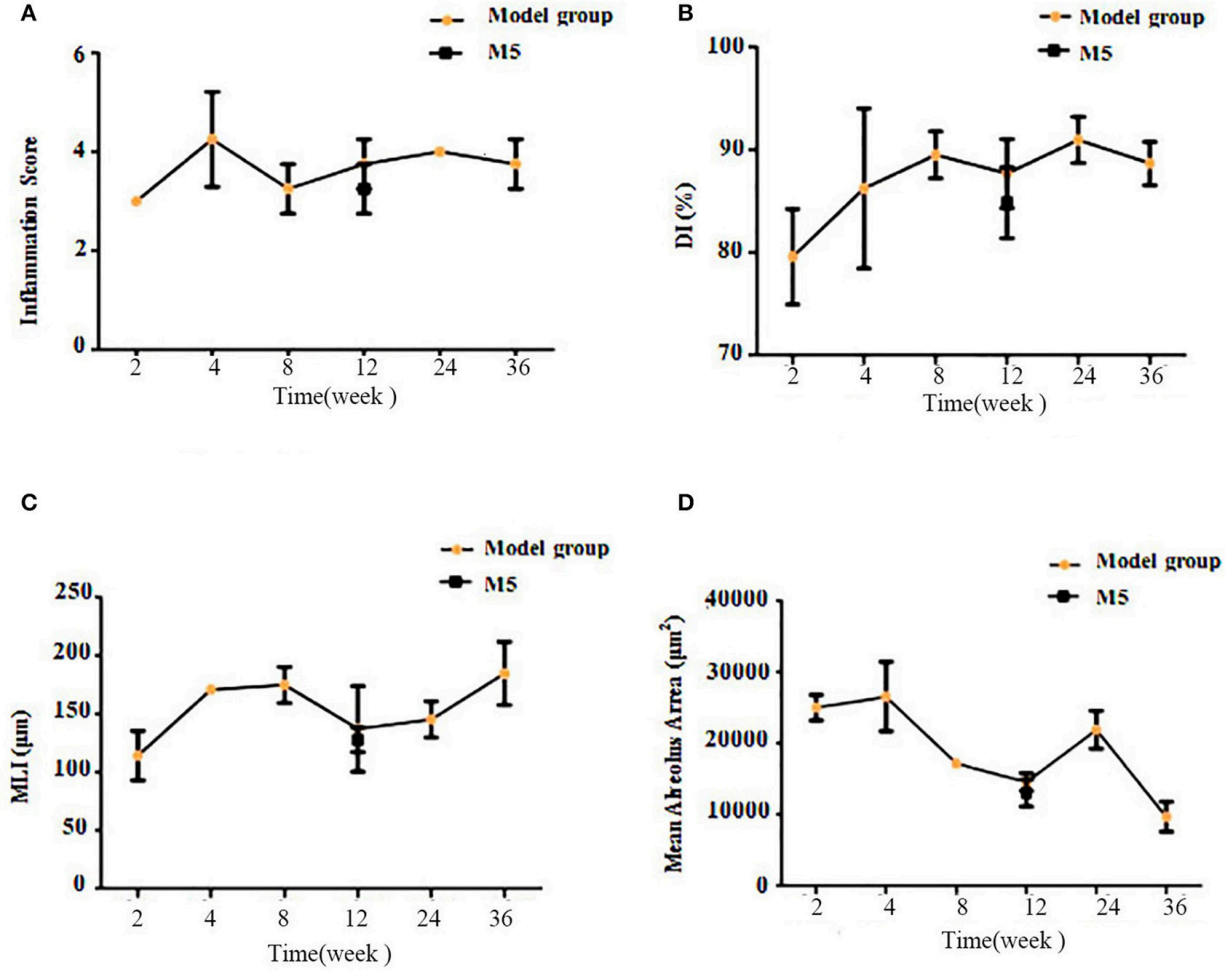
Comparison of lung tissue pathology findings between the model groups, based on semi-quantitative analysis of airway inflammation scores and emphysema indicators. **(A)** The airway inflammation score. **(B)** The destructive index (DI). **(C)** The mean linear intercept (MLI). **(D)** The mean alveolus area.

Based on the scope of inflammatory cell infiltration, the severity of inflammation was graded on a scale of 0–5 (see Lung Histopathology). Based on scale scores, inflammation increased to different levels after CS exposure, and the highest inflammation scores was seen in the 24-week CS group (Figure 5A). The DI represents the percentage of destroyed space as a fraction of the total alveolus and has high sensitivity in the determination of mild forms of emphysema (Saetta et al., 1985). Mean linear intercepts provided additional information about alveolar abnormalities, observed as air space enlargements (Robbesom et al., 2003). In the present study, DI and MLI were also changed to different extents and the highest levels were seen in the 24-week (Figure 5B) and 36-week CS groups (Figure 5C), respectively. The mean alveolus area showed a declining trend and decreased to the lowest levels in the 36-week CS group (Figure 5D). This finding indicates that CS exposure has a direct effect on airway inflammation, alveolar damage index, and MLI.

**Figure 6:**
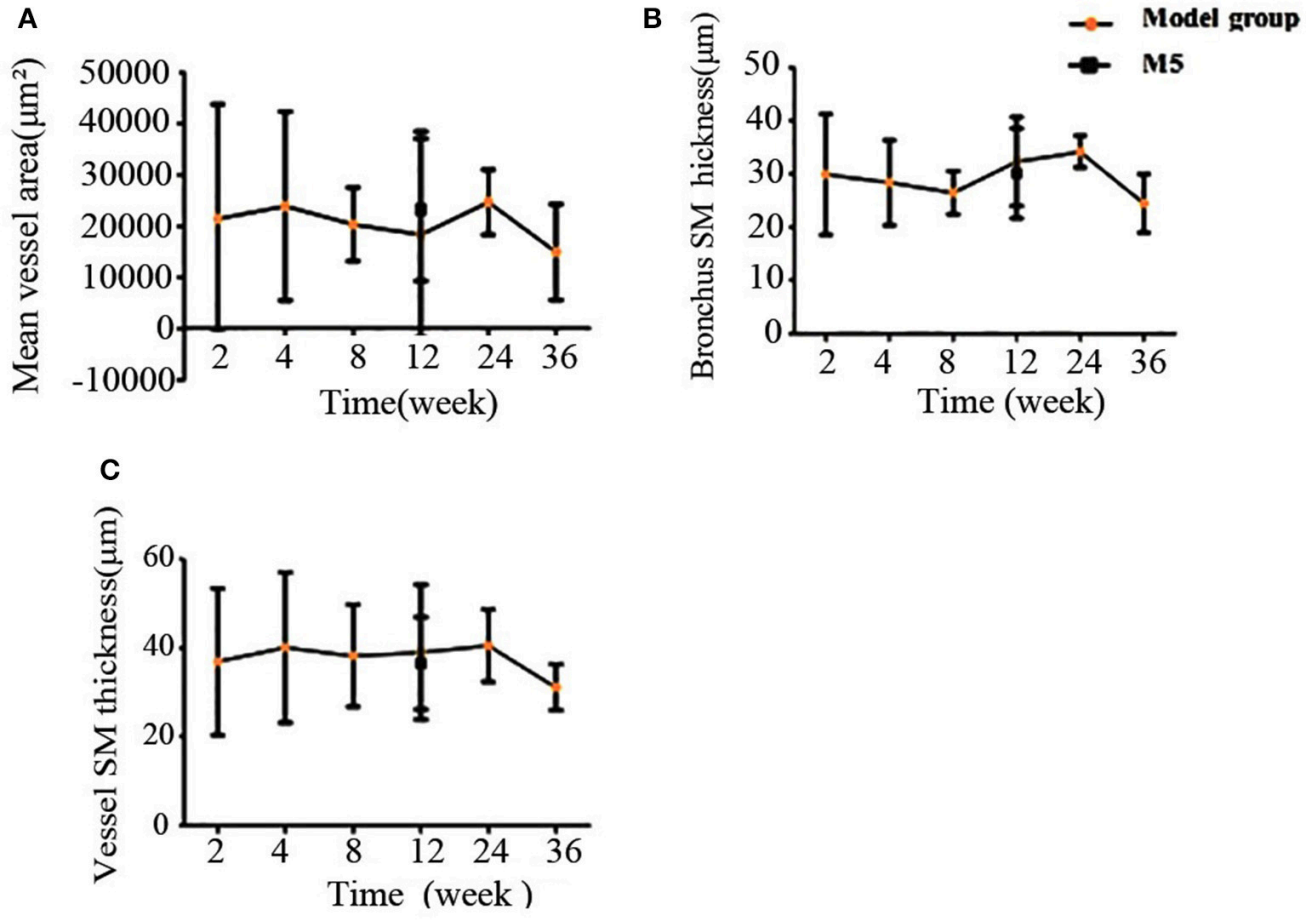
Comparison of HE-stained rat lung tissue of the model groups, based on semi-quantitative analysis. **(A)** Mean vessel area. **(B)** Bronchus smooth muscle thickness. **(C)** Vessel smooth muscle thickness. H&E, haematoxylin-eosin.

The mean vessel area, bronchus smooth muscle thickness, and vessel smooth muscle thickness were also quantified. As shown in Figure 6, these factors did not change to a comparable extent. However, the thickness of the bronchus smooth muscle and vessel smooth muscle increased to their peak in the 24-week CS group. This finding indicated that, compared with other indices, the vascular proliferation and airway remodeling-related indices have a weak association with CS exposure length (Figures 6A–C).

### Open-field test analysis

**Figure 7:**
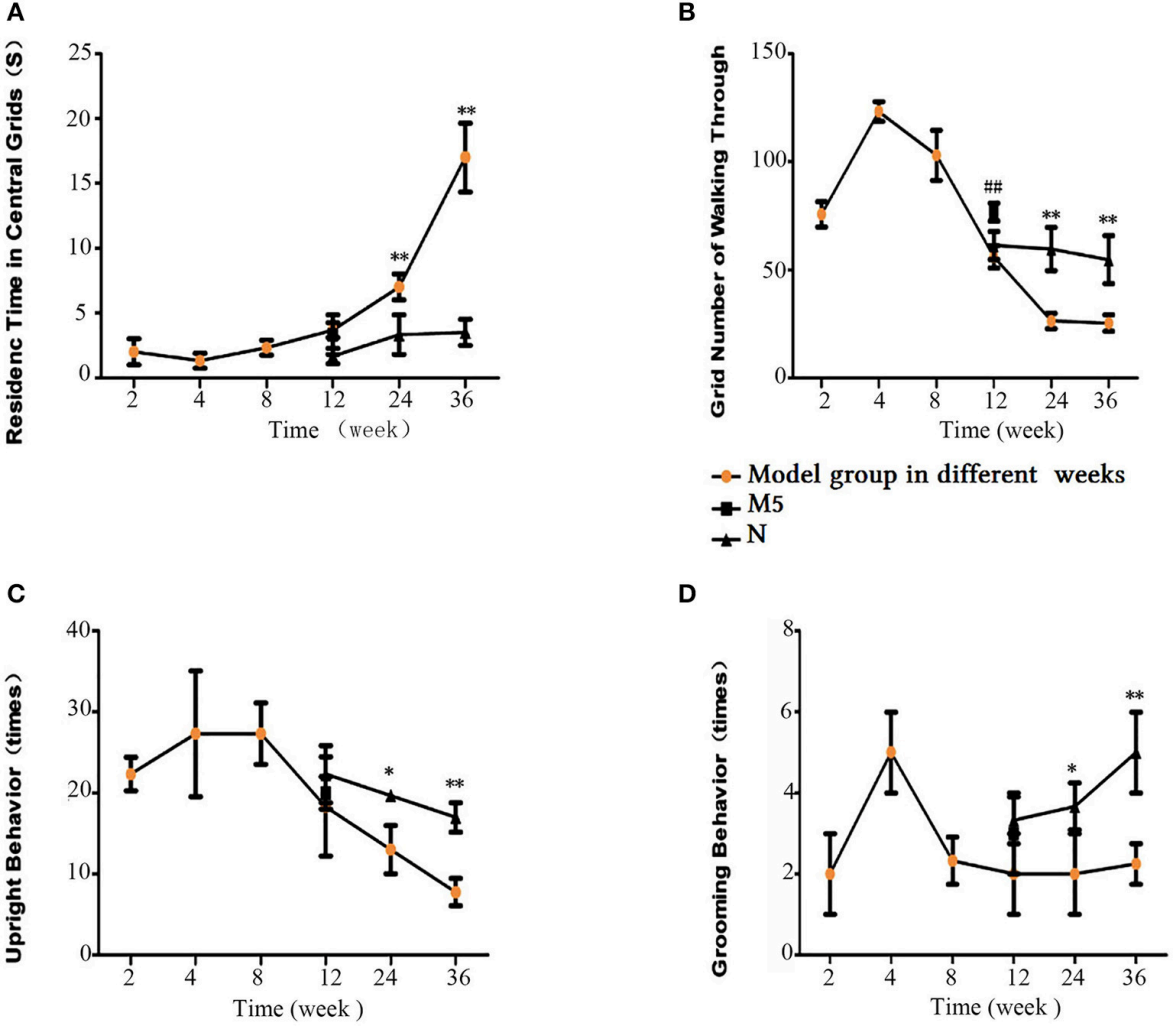
Open-field test results. **(A)** Residence time in the central grids. **(B)** Number of cross-links. **(C)** Upright grooming behavior. **(D)** Grooming Behavior. The data are presented as the mean ± the standard error of the mean (SEM). ^*^p < 0.05 and ^**^p < 0.01 indicate a statistically significant difference compared to the normal group. #*p* < 0.05 and ##*p* < 0.01 indicate a statistically significant difference between the M5 group and the M4 group.

The Global Initiative for Chronic Obstructive Lung Disease Guidelines 7 recommend that newly diagnosed COPD patients should be assessed for depression and anxiety, and social problems should be discussed. In addition, it has been reported that, as the respiratory manifestations of COPD progress, patients decrease their physical activity to avoid the discomfort of breathlessness (Hassett et al., 2014; Lee et al., 2017). In the 36-week CS exposure group, the rats spent the longest time staying in the center of the grid, and rearing and line-crossing frequency was the lowest. There was no significant difference between the 12-week CS exposure group (i.e., M4 group) and normal group (i.e., N1 group; *p* > 0.05). Significant differences were found between the 24- and 36-week CS exposure model groups and their respective control groups (i.e., M6 to N2 and M7 to N3). After 4 weeks of CS exposure cessation, the difference in the line crossing frequency between the M5 and M4 groups was significant (*p* < 0.01). Other indices also improved to some degree. Thus, general activity increased in the M5 group after stopping CS exposure compared with the model group without CS cessation (Figure 7). These findings indicate the importance of smoking cessation in the treatment of COPD.

### Blood gas analysis

**Figure 8:**
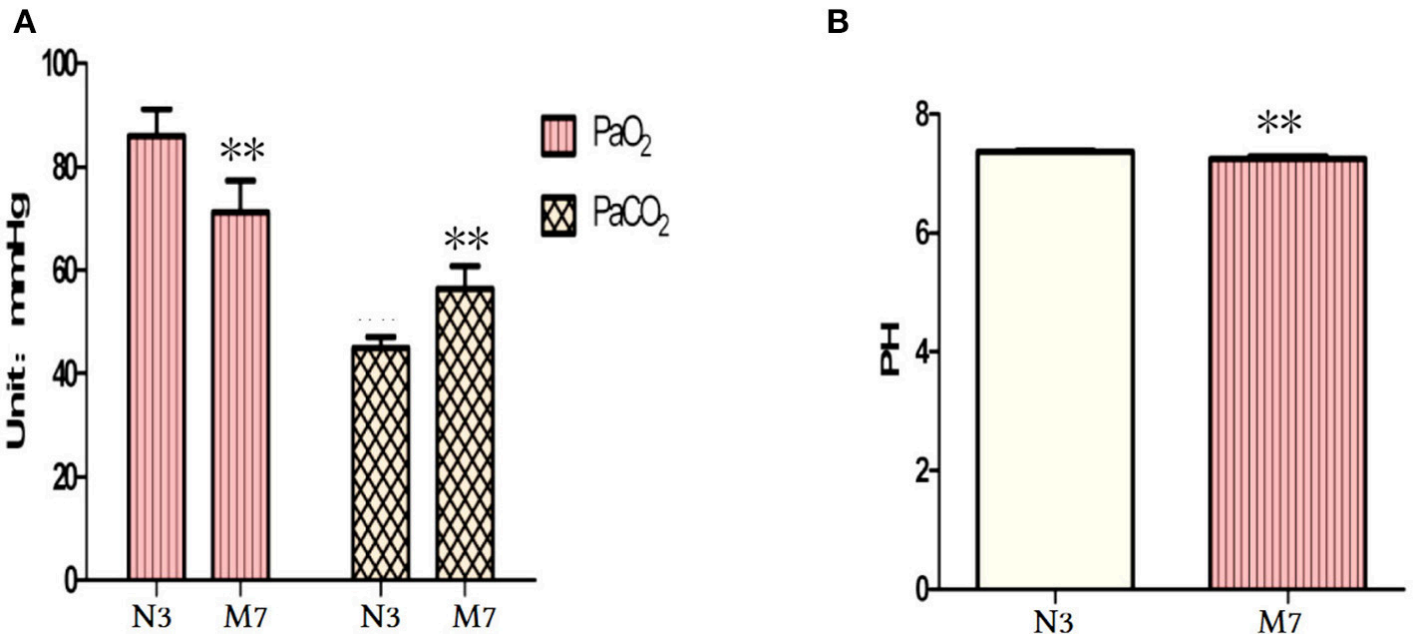
Blood gas analysis after 36 weeks of cigarette smoke (CS) exposure. **(A)** Arterial PaO_2_ and PaCO_2_ levels. **(B)** Blood pH. PaCO_2_, partial pressure of carbon dioxide; PaO_2_, partial pressure of oxygen. The data are presented as the mean ± the standard error of the mean (SEM). ^*^p < 0.05 and ^**^p < 0.01 indicate a statistically significant difference compared to the normal group.

There was little change in blood pH, arterial partial pressure of oxygen (PaO_2_), and arterial partial pressure of carbon dioxide (PaCO_2_) levels of the CS groups up to 24 weeks CS exposure (data not shown). However, in the 36-week CS exposure group, PaO_2_ significantly decreased and PaCO_2_ significantly increased compared with controls (*p* < 0.01, Figure 8A). Furthermore, blood pH was lower in the 36-week CS exposure group (*p* < 0.01, Figure 8B). These results indicate hypercarbia and hypoxaemia and an acid–base imbalance in the long-term CS exposure group. This finding is in concordance with the clinical features of patients with advanced-stage COPD.

### Inflammatory factors in blood serum

**Figure 9:**
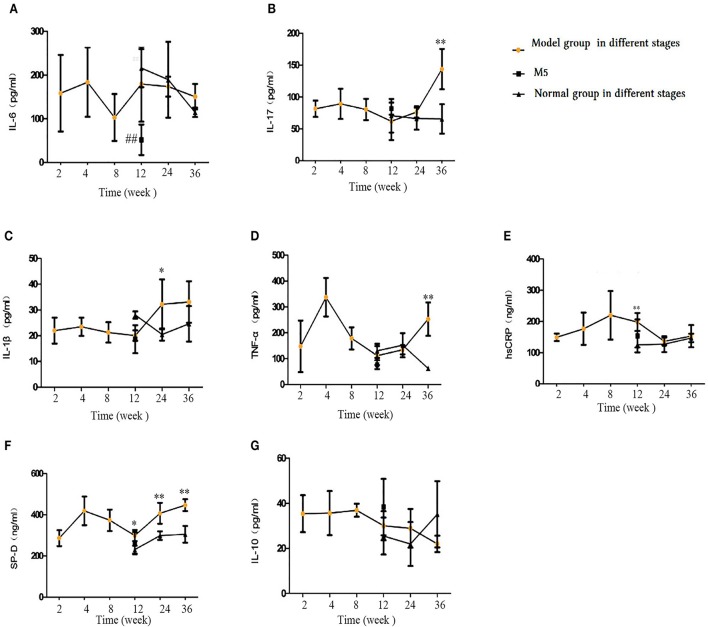
Comparison of inflammatory and anti-inflammatory factors in blood serum. **(A)** Interleukin (IL)-6. **(B)** IL-17. **(C)** IL-1β. **(D)** Tumor necrosis factor (TNF)-α. **(E)** IL-10. **(F)** surfactant protein D (SP-D), **(G)** Interleukin(IL)-10. The data are expressed as the mean ± standard deviation. ^*^p < 0.05 and ^**^p < 0.01 indicate a statistically significant difference compared to the normal group. ^#^*p* < 0.05 and ^##^*p* < 0.01 indicate a statistically significant difference between the M5 group and the M4 group.

As shown in Figure 9, in the early CS exposure period, some inflammatory factors showed an upward trend. after 36 weeks of CS exposure group, we saw a marked increase in TNF-a, IL-17, and surfactant protein D (SP-D) levels (*p* < 0.05 or *p* < 0.01, Figures 9B,D,F) in the blood serum, whereas no change was observed in IL-6, IL-10, or high-sensitivity C-reactive protein (hsCRP) levels (*p* > 0.05, Figures 9A,G,E). IL-β1 and SP-D levels also showed a significant increase in the 24-weeks CS exposure group, compared with that in the control group. In the 12-week CS group, hsCRP, a proxy for the inflammatory response, and SP-D increased significantly compared with the control group (*p* < 0.05 or *p* < 0.01). On comparing the M5 and M4 groups, no differences were found in the levels of IL-17, IL-1β, TNF-α, HcCRP, SP-D, or IL-10 (*p* > 0.05), but IL-6 displayed significant decrease (*p* < 0.05, Figure 9A). This finding indicates that stopping CS exposure even short-term can partially regulated the proinflammatory and anti-inflammatory balance.

### Change in oxidative stress related factors in blood serum

**Figure 10:**
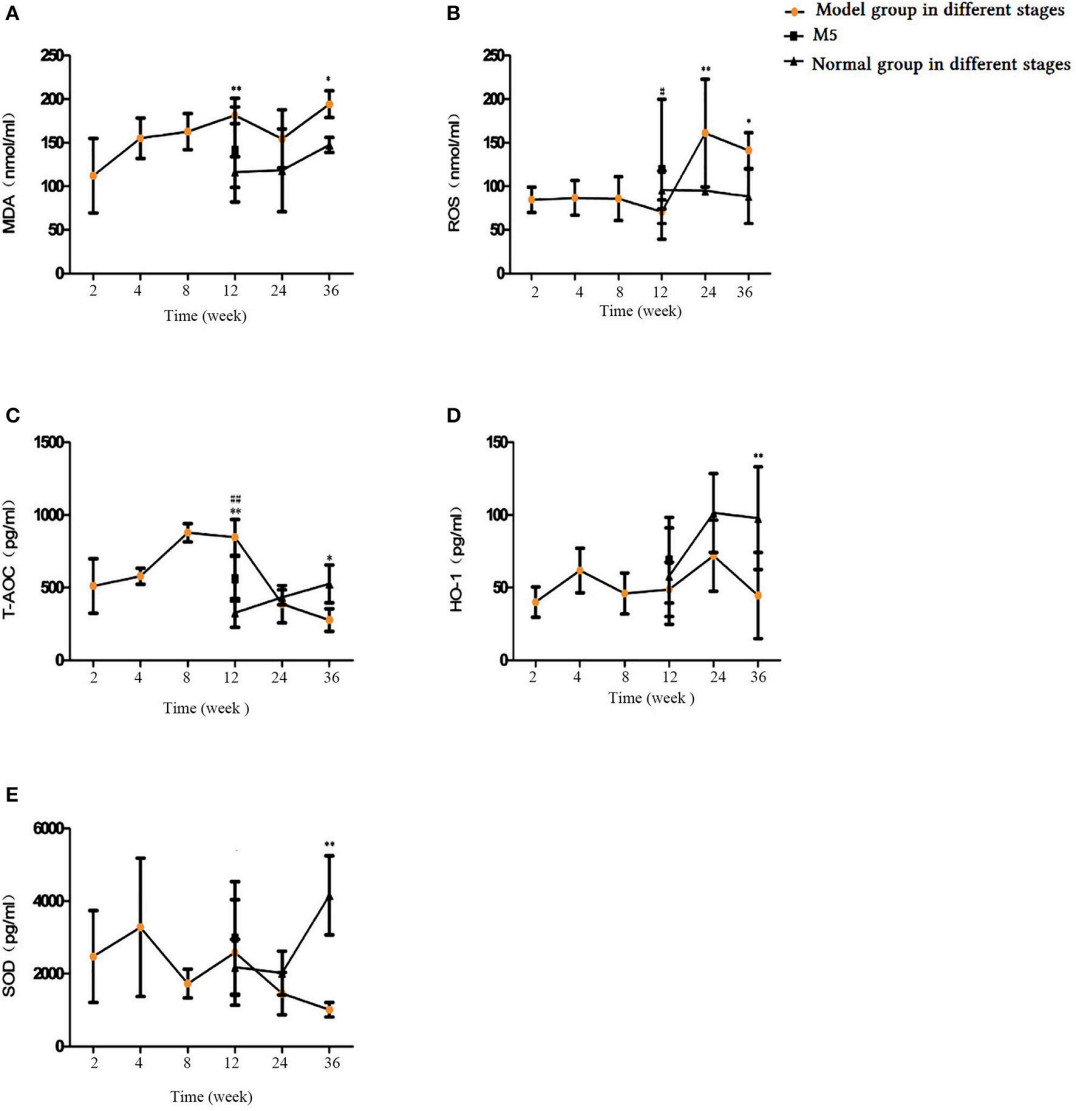
Comparison of oxidative and antioxidative factors in blood serum. **(A)** Malondialdehyde (MDA), **(B)** Reactive oxygen species (ROS), **(C)** Total antioxidant capacity (T-AOC), **(D)** Heme oxygenase-1 (HO-1), **(E)** Superoxide dismutase (SOD). The data are expressed as the mean ± standard deviation. ^*^p < 0.05 and ^**^p < 0.01 indicate a statistically significantly difference compared to the normal group. ^#^*p* < 0.05 and ^##^*p* < 0.01 indicate a statistically significant difference between the M5 group and the M4 group.

Our examination of blood serum oxidative and antioxidative factors showed that the MDA and reactive oxygen species (ROS) levels increased as the CS exposure time increased; the 24- and 36- CS exposure groups displayed significantly higher ROS levels than did the control groups (*p* < 0.05, *p* < 0.01, respectively; Figure 10B), whereas MDA levels were markedly higher in the 36-week CS group alone (*p* < 0.05, Figure 10A). The antioxidative indicators total antioxidant capacity, HO-1 and SOD decreased initially and then increased by the final time point; they reached their lowest level at week 36 (Figures 10C–E). Compared with the M4 group, ROS and total antioxidant capacity (T-AOC) were decreased significantly in the M5 group (*p* < 0.05), while no changes were observed in SOD, HO-1, or MDA levels (*p* > 0.05). This demonstrates that, in the early stages of CS exposure, the oxidation and antioxidation capabilities of rats primarily rely on the animal's ability to relieve stress. With the increase in CS exposure, antioxidative capability at 24 weeks was significantly decreased and resulted in an oxidative–antioxidative imbalance.

### Airway remodeling factors in blood serum

**Figure 11:**
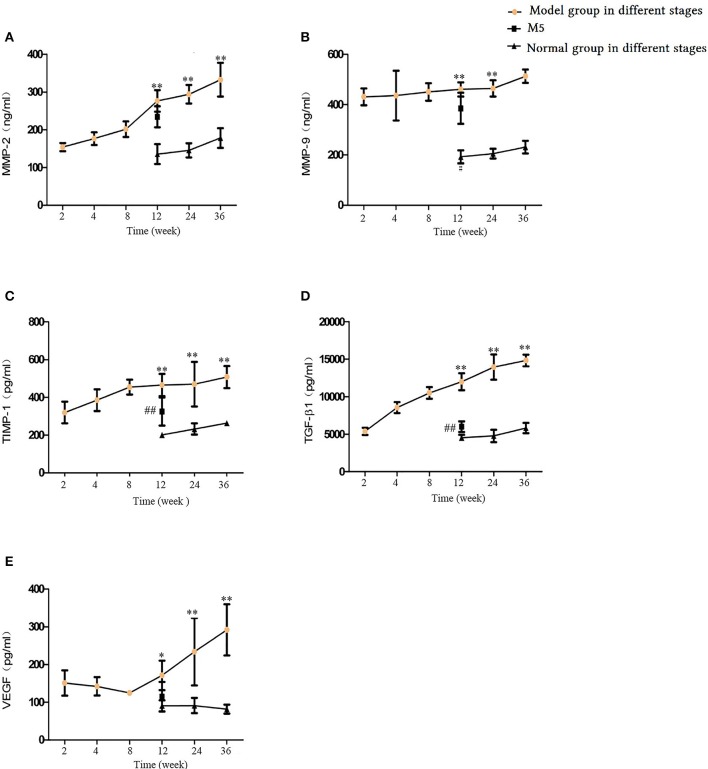
Comparison of airway remodeling factors in blood serum. **(A)** Matrix metalloproteinase (MMP)-2. **(B)** MMP-9. **(C)** Tissue inhibitor of metalloproteinase 1 (TIMP-1). **(D)** Transforming growth factor beta-1 (TGFβ-1). **(E)** Vascular endothelial growth factor (VEGF). The data are expressed as the mean ± standard deviation. ^*^p < 0.05 and ^**^p < 0.01 indicate a statistically significant difference compared to the normal group. ^#^*p* < 0.05 and ^##^*p* < 0.01 indicate a statistically significant difference between the M5 group and the M4 group.

To delineate the effects of long-term CS exposure on airway remodeling, we investigated related factors in the blood serum. With increasing CS exposure time, the levels of MMP-2, MMP-9, TIMP-1, TGF-β1, and vascular endothelial growth factor were significantly higher in the M4, M6, and M7 groups compared with the normal groups (*p* < 0.05 and < 0.01; Figures 11A–E), and reached the highest level after 36 weeks of CS exposure. This finding indicates that CS exposure elevates rat protease and antiprotease factors and, thereby, results in an imbalance. Compared with the M4 group, after 4 weeks of CS cessation in the M5 group, TGF-β1, MMP-9, and TIMP-1 levels were significantly decreased (*p* < 0.05 and < 0.01, Figures 11B,C,E).

### Pro-inflammatory and anti-inflammatory factors in BALF

**Figure 12:**
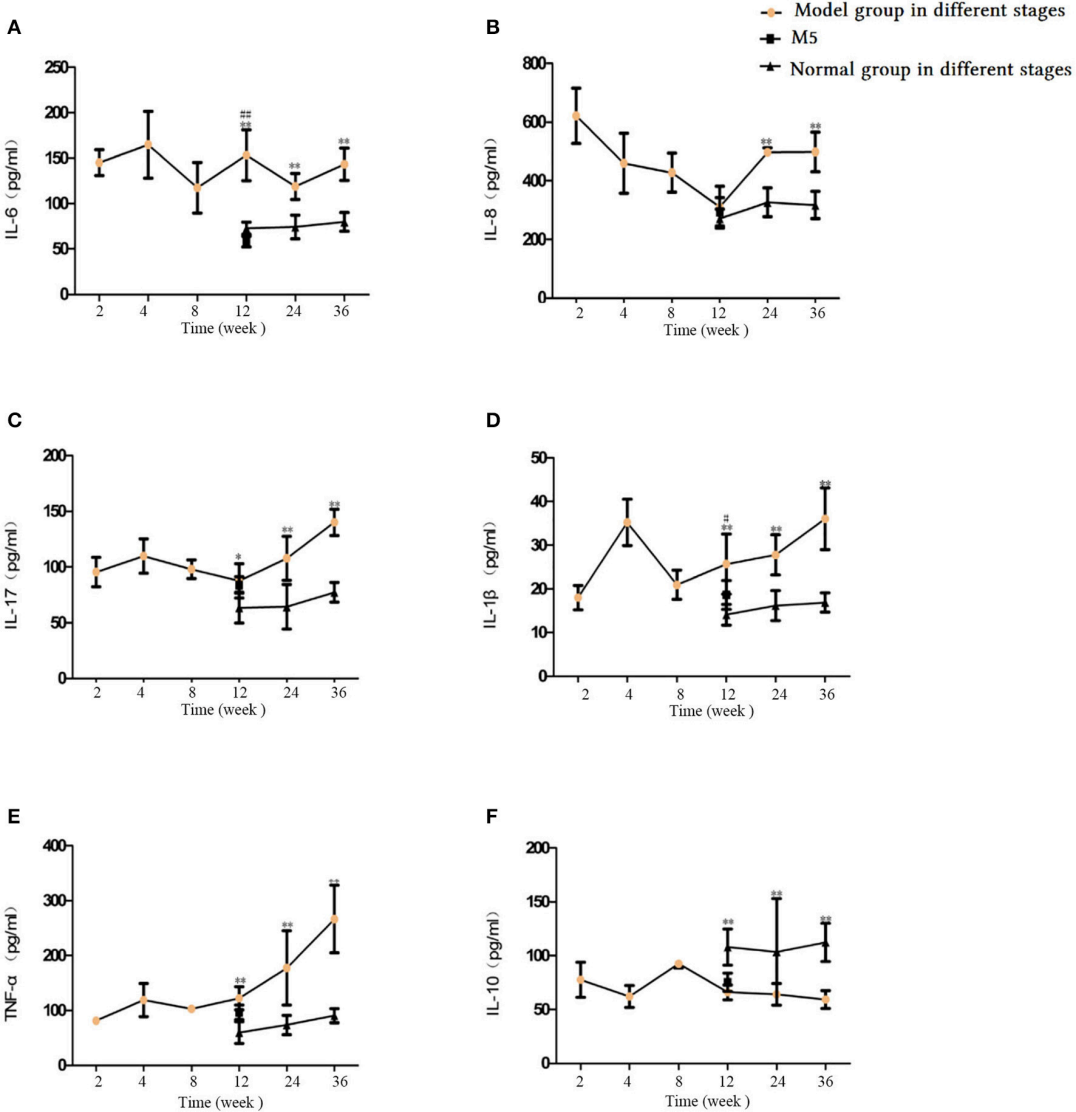
Comparison of inflammatory and anti-inflammatory factors in bronchoalveolar lavage fluid (BALF). **(A)** Interleukin (IL)-6. **(B)** IL-8. **(C)** IL-17. **(D)** IL-1β. **(E)** Tumor necrosis factor (TNF)-α. **(F)** IL-10. The data are expressed as the mean ± standard deviation. ^*^p < 0.05 and ^**^p < 0.01 indicate a statistically significant difference compared to the normal group. ^#^*p* < 0.05 and ^##^*p* < 0.01 indicate a statistically significant difference between the M5 group and the M4 group.

Our investigation of the levels of IL-6, IL-8, IL-17, IL-1, TNF-α, and IL-10 in BALF showed that, in the 12-, 24-, and 36-week CS exposure groups, the IL-6, IL-17, IL-1β, and TNF-α levels were significantly higher, while IL-10 levels were decreased (*p* < 0.05 or *p* < 0.01, Figures 12A,C–F). IL-8 levels were also elevated after 24 and 36 weeks of CS exposure (Figure 12B). In a comparison of M5 and M4 groups, IL-6, IL-1β, IL-17, and TNF-α levels were lower after 4 weeks of smoke exposure cessation, whereas IL-10 levels were significantly higher (*p* < 0.05 or < 0.01, Figures 12A,C,E,F). By comparing the M5 and M4 groups, we found that IL-6, IL-1β, IL-17, and TNF-α levels were decreased to varying degrees and that IL-10 levels were elevated (*p* < 0.05 or *p* < 0.01, Figures 12A,C,E,F). The inflammatory indicators in BALF showed the same patterns as those in the blood serum.

### Effects of CS on pro-inflammatory and anti-inflammatory factors in lung tissue homogenate

**Figure 13:**
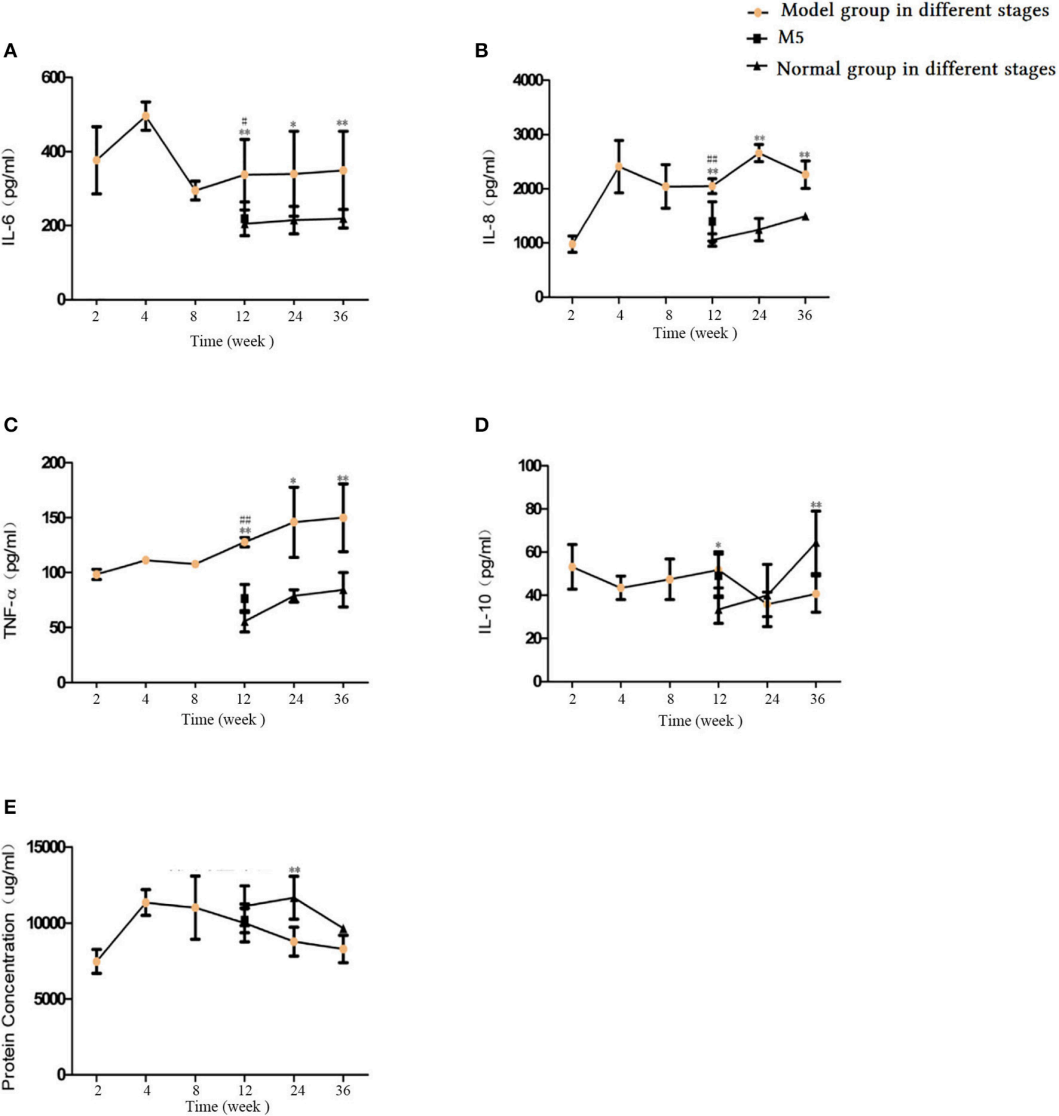
Comparison of inflammatory and anti-inflammatory factors in lung tissue. **(A)** Interleukin (IL)-6. **(B)** IL-8. **(C)** Tumor necrosis factor (TNF)-α. **(D)** IL-10. **(E)** Protein concentration. The data are expressed as the mean ± standard deviation. ^*^p < 0.05 and ^**^p < 0.01 indicate a statistically significant difference compared to the normal group. ^#^*p* < 0.05 and ^##^*p* < 0.01 indicate a statistically significant difference between the M5 group and the M4 group.

As shown in Figure 13, IL-6, IL-8, and TNF-α levels in the model groups' lung tissue were significantly higher at weeks 12, 24, and 36 compared with their levels in the corresponding normal groups (*p* < 0.05 or *p* < 0.01, Figures 13A–C), whereas lower levels were observed for IL-10 levels at 12 and 36 weeks of CS exposure (*p* < 0.05 or < 0.01, Figure 13D). IL-6, IL-8, and TNF-α levels were significantly lower in the M5 group than in the M4 group (*p* < 0.05, Figures 13A–C). These factors showed the same variation in the blood serum and BALF. This finding demonstrates that stopping CS exposure can attenuate inflammation in lung tissue to some degree. In addition, pro-inflammatory factors (i.e., IL-6 and IL-10) in the M2 group increased to their peak point, whereas the anti-inflammatory factor IL-10 decreased to its lowest point, which reflected an inflammatory feature at 4 weeks of CS exposure. The protein content in the lung tissue in the M1 and M2 groups displayed an upward trend, reached a peak at week 4, and then showed a downward trend to week 36 (Figure 13E).

## Discussion

An ideal animal model must first be characterized and demonstrated as being an appropriate model for a particular form of a human disease or reproduce conditions found in the original species (Coffey and Isaacs, 1980; Davidson et al., 1987). To date, COPD models have been established using various species, such as rodents, monkeys, sheeps, dogs, guinea pigs, (Ghorani et al., 2017). However, none are exact mimics of the human situation, and the appropriateness of a model highly depends on the study aims. There is some controversy on the establishment of a COPD rat model. It was reported that a rat model is a poor animal model because rats are relatively resistant to COPD model establishment (Groneberg and Chung, 2004; Stevenson et al., 2007). Conversely, another study reported that the rat is a favorable and widely used model and that with only 2 months of smoke exposure, emphysematous changes that further progress can be measured (Leberl et al., 2013) and various reports have been written using a COPD rat model. However, each choice of an animal model has its own benefits and shortcomings. In addition, humans are not very susceptible to COPD; it takes years for humans to develop COPD because of CS. Thereby, rats may be one of the favored options for mimicking the course of COPD development because of its larger size, facilitated experimental interventions, and genomic and behavioral features (Coffey and Isaacs, 1980; Jacob, 1999). Various cigarette types and exposure methods were also used to establish COPD model (Cendon et al., 1997; Zheng et al., 2009; Liu et al., 2010). These results show that different methods work for the induction of COPD in rats, varying based on the cigarette type, duration, and exposure method. In the present study, we used 3R4F research cigarettes produced by The University of Kentucky, with nicotine and tar content lower than domestic cigarettes. Typically, standard research-grade cigarettes are useful to deliver a specified dose of total suspended particles or total particulate matter, including nicotine and carbon monoxide (Leberl et al., 2013; Ghorani et al., 2017). Recently, several COPD mouse models have been reported with different exposure period (Takubo et al., 2002; Stevenson et al., 2005), but the number of cigarettes for CS induction was not mentioned; the exposure period is unclear. A COPD model that exposed mice to 3 cigarettes/d, 5 d/week for 7 months was also reported, but lung function was not evaluated (Martorana et al., 2005). A sidestream CS-induced rat model was also reported with chronic features of COPD using double happiness brand cigarettes with a detailed analysis of lung function. However, the research cigarette was not used, which is controlled in total particular matter content and also further evidence of their which support model establishment was not reported (Zheng et al., 2009). The present study has several advantages compared with other CS animal models in that it evaluates overall COPD-related indicators, including lung function, histopathology, open-field test, blood gas, and cytokine levels in blood serum, BALF, and lung tissue, which are indicators of successful COPD models. We used multiple groups with 2–36 weeks of six cigarettes per day and also included an 8-week CS exposure with 4 weeks of CS cessation group, because it was reported that chronic period starts at 8 weeks of CS exposure in rats (Kratzer et al., 2013). We evaluated the effects of passive smoking on the establishment of a COPD model and also the effects of quitting smoking on COPD exacerbation. With respect to the convenient observations and comparisons, we designed a normal control group to match with each of the CS groups so as to increase the comparability and reliability of the study. Our results indicate that animals receiving 24 and 36 weeks of CS exposure satisfied the following requirements for a successful COPD animal model: firstly, pathological changes (Wright and Churg, 2002), including goblet epithelium cells metaplasia, evident epithelial cells injury of bronchi, infiltration of inflammatory cells into the bronchiolar wall (Chunhua et al., 2017), mucus hypersecretion and secretion (Barnes et al., 2003), and cell blocking in the bronchial lumen (Zhou et al., 2016) and second, the presence of non-normalizing airflow limitations (Hassett et al., 2014), such as airway narrowing compared with a normal airway (Wiggs et al., 1992), the wall internal to the muscle was significantly thickened. Moreover, a rapid decline of FEV1, and a reduction in the ratio of FEV1 to forced VC (Tashkin et al., 1996; Chung, 2005) was also seen.

Previous studies showed that various inflammatory factors and chemokines also play an important role in the development of COPD and inflammatory mediators, such as IL-1β, IL-6, IL-8, and TNF-α, amplify the inflammatory response, while anti-inflammatory factors, such as IL-10, increase to cope with the airway inflammation (Zhou et al., 2016).

MMPs play a key role in airway remodeling, inflammation, and emphysema (Lagente and Boichot, 2010). It has been reported that levels of MMP-1, MMP-2, and MMP-9 are increased in COPD patients, (Cataldo et al., 2000; Imai et al., 2001; Kistemaker et al., 2012 #89). MDA is thought to be as a marker useful in evaluating the degree of lipid peroxidation pulmonary dysfunction (Jing et al., 2015). SOD is responsible for removing oxyradicals in cells, whose activity may represent the degree of tissue damage. In the present study, inflammatory, airway remodeling/anti-remodeling, and oxidative stress related factors were observed in blood serum, BALF, and lung tissue, and our results show that inflammatory factors maintained the same upward and downward trends in serum, BALF, and lung tissue. The 24- and 36-week CS exposure groups demonstrated moderate- and advanced-stage inflammatory responses. Blood gas analyses are important indicators of respiratory function (Hassett et al., 2014). In patients with severe COPD, arterial blood gas analysis can indicate a life-threatening episode that needs close monitoring or intensive care (Wang et al., 2017; Zhai et al., 2017). In the present study, the blood gas analysis results suggested that the blood gas analysis indices were significantly higher in the CS exposure groups than in the control groups, and these animals displayed advanced-stage clinical features of COPD patients.

The only effective intervention to slow down the accelerated decline in lung function in smokers with chronic obstructive pulmonary disease is stop smoking (van Eerd et al., 2017). Previous clinical studies reported that in COPD patients, smoking cessation is associated with life quality improvement, those COPD patients with low quality of life get the biggest benefit from quitting smoking, but in aging patients this difference diminished (Jimenez-Ruiz et al., 2017). In our study, in order to evaluate the effects of stop second hand smoke inhalation on COPD rats which were exposed to room air 4 weeks for recovery after 8 weeks of side stream smoke exposure. Our results showed that stop side stream smoke inhalation parly improved lung function and some inflammatory factors declined compared with counterpart 12 week CS exposure group. These results are consistent with previous clinical reports and indicated the importance of stop second hand smoke inhalation for improving the life quality of passive smokers.

In conclusion, our results showed that 24 and 36 weeks of sidestream CS exposure (6 cigarettes per day) produces a COPD rat model that is suitable for the observation of moderate- and advanced-stage COPD pathogenesis, and we provide here the first line of evidence on the relationship between passive smoking and COPD. The results herein also demonstrate the importance of smoking cessation the course of COPD treatment.

## Ethics statement

All animals were handled in strict accordance with the relevant national and local animal welfare bodies, and all experiment was approved by the institutional review board of Shanghai medical college of Fudan University (permit number: SYXK(hu)2010–0099) and in accordance with the guidelines for animal use of national institutes of health, China.

## Author contributions

GW, JS, YL, HJ, and JL performed the animal model-making of COPD. LK performed the Lung function evaluttion, Open field test. ZZ performed pulmonary histopathologyanalysis. NM wrote the manuscript. HZ, JD, and NM analyzed the data and revised the manuscript. All the authors read and approved the final manuscript, and agree to be accountable for all aspects of the work. GW and NM contributed equally to this work.

### Conflict of interest statement

The authors declare that the research was conducted in the absence of any commercial or financial relationships that could be construed as a potential conflict of interest.

## Abbreviations

ABG: arterial blood gas
BALF: bronchoalveolar lavage fluid
COPD: chronic obstructive pulmonary disease
CS, cigarette smoke DI: destructive index
FRC: functional residual capacity
FVC: forced vital capacity
H&E: haematoxylin–eosin
MLI: mean linear intercept
PA: physical activity
ROS: reactive oxygen species
SOD: superoxide dismutase
TLC: total lung capacity
VC: vital capacity
VEGF: vascular endothelial growth factor.
